# Nucleolus and Nucleolar Stress: From Cell Fate Decision to Disease Development

**DOI:** 10.3390/cells11193017

**Published:** 2022-09-27

**Authors:** Lu Hua, Daliang Yan, Chunhua Wan, Baoying Hu

**Affiliations:** 1 Department of Oncology, Taizhou People’s Hospital, Taizhou 225300, China; 2 Department of Cardiovascular Surgery, Taizhou People’s Hospital, Taizhou 225300, China; 3 School of Public Health, Nantong University, Nantong 226001, China; 4 Department of Immunology, Medical College, Nantong University, Nantong 226001, China

**Keywords:** nucleolus, nucleolar stress, ribosome biogenesis, p53, senescence, apoptosis, autophagy, cancer, neurodegenerative disease

## Abstract

Besides the canonical function in ribosome biogenesis, there have been significant recent advances towards the fascinating roles of the nucleolus in stress response, cell destiny decision and disease progression. Nucleolar stress, an emerging concept describing aberrant nucleolar structure and function as a result of impaired rRNA synthesis and ribosome biogenesis under stress conditions, has been linked to a variety of signaling transductions, including but not limited to Mdm2-p53, NF-κB and HIF-1α pathways. Studies have uncovered that nucleolus is a stress sensor and signaling hub when cells encounter various stress conditions, such as nutrient deprivation, DNA damage and oxidative and thermal stress. Consequently, nucleolar stress plays a pivotal role in the determination of cell fate, such as apoptosis, senescence, autophagy and differentiation, in response to stress-induced damage. Nucleolar homeostasis has been involved in the pathogenesis of various chronic diseases, particularly tumorigenesis, neurodegenerative diseases and metabolic disorders. Mechanistic insights have revealed the indispensable role of nucleolus-initiated signaling in the progression of these diseases. Accordingly, the intervention of nucleolar stress may pave the path for developing novel therapies against these diseases. In this review, we systemically summarize recent findings linking the nucleolus to stress responses, signaling transduction and cell-fate decision, set the spotlight on the mechanisms by which nucleolar stress drives disease progression, and highlight the merit of the intervening nucleolus in disease treatment.

## 1. Introduction

The nucleolus is a dynamic sub-nuclear compartment that plays a central role in ribosome biogenesis [1]. In mammalian cells, nucleoli are assembled around acrocentric chromosomal regions encoding clusters of ribosomal DNA (rDNA) repeats, termed nucleolar organizer regions (NORs). The human genome contains over 300 rDNA units distributed in five acrocentric chromosomes, namely, chr13, chr14, chr15, chr21 and chr22 [2]. A functional nucleolus is characterized by three distinct compartments: the fibrillar center (FC; marker protein RNA polymerase I subunit A (POLR1A) or upstream binding factor (UBF)), the dense fibrillar component (DFC; marker protein fibrillarin (FBL)) and the granular component (GC; marker protein nucleophosmin (NPM)) [3]. Ribosome biogenesis first takes place in FC, where rDNA is transcribed by RNA polymerase I (Pol I) transcriptional machinery into 47S rRNA. Then, nascent 47S rRNA is processed in DFC and GC, where mature 18 S, 5.8 S and 28 S rRNAs are generated by the cleavage of unnecessary transcribed spacers [4]. In addition, 5S rRNA is synthesized by RNA polymerase III (Pol III) in the nucleoplasm and translocated into the nucleolus. These rRNAs are assembled together with ribosomal proteins to generate 40S and 60S ribosomal subunits, followed by their exports from the nucleolus to the cytoplasm.

Ribosome biogenesis is an energy-consuming process that is well-orchestrated by intracellular signaling networks [5,6]. The dynamics and architecture of nucleoli are coupled with the need of ribosome biogenesis and the protein synthesis of cells [7]. For instance, nucleolar sizes and numbers of cancer cells may be indicative for their proliferative potential and aggressiveness [8]. In fact, the evaluation of the quantity of interphase nucleoli by silver-staining-mediated visualization of NORs (AgNORs) has been employed as a reliable approach to assess the prognosis of multiple cancer types [9,10]. Given the fact that nucleolar function is closely linked to proliferative status and metabolic activity of cells, it is unsurprising that nucleolar architecture may reflect the progression of assorted human diseases, particularly cancer development. Notably, besides its canonical role in ribosome biogenesis, studies in the past decades have uncovered that the nucleolus is a crucial hub for signaling transduction and stress response, underscoring the importance of nucleolus-initiated signals as important drivers of disease progression.

Mounting data have revealed a pivotal role of nucleolar physiology in disease development, including tumorigenesis, neurodegenerative diseases and aging [11]. The involvement of the nucleolus in disease development is largely attributed to its role as a sensing hub of stress signaling [12]. In response to a wide range of stress stimuli, nucleolar stress is initiated as a result of aberrant ribosome biogenesis and nucleolar dynamics [13,14]. As a consequence, nucleolar stress facilitates various signaling transductions, including those fundamental to cell fate decision and disease progression. In this review, we discuss the molecular mechanisms linking nucleolar physiology and stress to aberrant signaling and disease development, highlight the importance of nucleolar stress as an integral part of the intracellular signaling network and emphasize nucleolar intervention as a promising option for disease prevention and therapy.

## 2. Dynamic Regulation of Nucleolar Architecture

The dynamics of nucleolar architecture is closely related with rRNA transcription and ribosome biogenesis. Mammalian oocytes and early embryos contain inactive nucleolus precursor bodies (NPBs), characterized by the presence of ribosomal proteins, fibrillarin and heterogeneous nuclear ribonucleoproteins (hnRNPs) [15]. The initiation of the nucleolar assembly occurs at the late 2-cell stage, coinciding with the activation of Pol I-mediated rRNA transcription [16]. The expression of rRNA may serve as a seeding mechanism to trigger the recruitment of nucleolar proteins, whose nucleolar localization sequences (NoLS) contain a high proportion of positively charged amino acids and may easily bind to negatively charged rRNA [17]. In the absence of rRNA, nucleolar proteins may form highly variable protein aggregates that are distinct from normal nucleolar architecture, suggesting an indispensable role of rRNA transcription in the nucleolar assembly [18].

Recent studies have indicated that the nucleolus is assembled through liquid–liquid phase separation (LLPS) [3,19]. Members of core nucleolar components may self-assemble with rRNA through heterotypic interactions among nucleolar proteins and RNA, whereas some others may be actively recruited by the nucleolus [19,20]. Proteins may be dynamically accumulated within nucleoli and constitutively shuttle between the nucleoplasm and the nucleolus. The shuttling of multiple nucleolar proteins, such as NPM, is coupled with ribosome export, and the blockade of NPM shuttling causes deficits in ribosome export and protein synthesis [21]. The disruption of Pol I-mediated rRNA transcription may cause aberrant nucleolar dynamics, leading to aberrant shuttling and localization of nucleolar proteins. Accordingly, the regulation of Pol I activity by various upstream regulators plays a crucial role in nucleolar dynamics and distribution of nucleolar proteins.

rRNA transcription and processing involves the recruitment of Pol I transcriptional machinery and the subsequent RNA splicing and maturation, followed by the assembly and export of ribosome subunits. Pol I transcriptional machinery is composed of Pol I core subunits and multiple essential transcriptional co-factors, such as TIF-IA, UBF and SL-1, whose availability and activity are of great importance to the regulation of rRNA transcription under various physiological and stress conditions [22]. The initiation of rRNA transcription requires the binding of the UBF/SL-1 preinitiation complex on the rDNA promoter [23,24]. The preinitiation complex then facilitates the assembly of Pol I transcriptional machinery by recruiting Pol I, TIF-IA and other transcriptional co-factors, such as Sirt7 and Casein Kinase II (CK2) [25,26]. The assembly of Pol I transcriptional machinery also involves post-transcriptional modifications on the core subunits, such as Pol I, TIF-IA and UBF, upon various upstream signals [27,28]. The transcribed 47S rRNA is then subjected to splicing and chemical modifications with the assistance of multiple RNA processing proteins, such as splicing factors, RNA helicases and small nucleolar RNAs (snoRNAs) [29,30,31]. Subsequently, the mature rRNAs, together with 5S rRNA and ribosomal proteins, were assembled into 40S and 60S ribosome subunits, followed by their nucleolar export.

## 3. Coordinate Regulation of Pol I-Mediated rRNA Transcription

**Figure 1:**
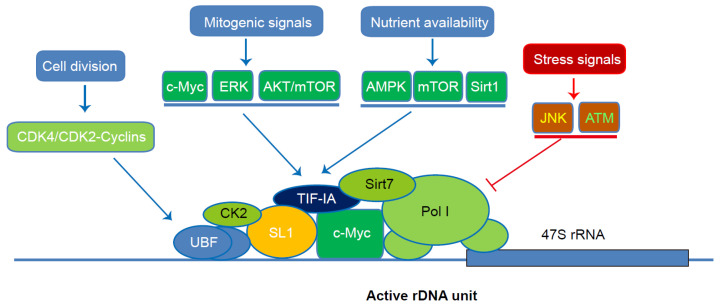
Coordinate regulation of Pol I-mediated transcription of rRNA by upstream signaling pathways. Multiple components of Pol I transcriptional machinery, such as UBF, SL1, TIF-IA and Pol I, are subjected to post-transcriptional modifications by various upstream regulators. These regulations coordinate the requirement of rRNA synthesis to cellular demand for ribosome biogenesis.

While the transcription of rRNA plays a fundamental role in nucleolar dynamics and architecture, Pol I-mediated transcription itself is fine-tuned by the cell physiological state. Because of its central role in ribosome biogenesis and protein translation, delicate regulation of rRNA transcription is critical for the transition of cell physiology, including cell proliferation, differentiation and stress responses [32,33]. This requires precise regulation to translate the demand of ribosome biogenesis into Pol I activation and rRNA transcription [34]. In this regard, mounting studies have revealed that rRNA transcription is tightly regulated by signaling pathways in response to various upstream stimuli (Figure 1).

First and foremost, growth-related signals are of vital importance in the regulation of Pol I activity and rRNA transcription. In this regard, c-Myc serves as a master regulator linking mitogenic signals to Pol I-initiated transcription (recently reviewed by Isabella Brown and colleagues) [35]. c-Myc may bind to rDNA and recruit Pol I machinery to drive the transcription of rRNA in response to mitogenic signals [36,37]. In addition to c-Myc, multiple mitogenic kinase cascades are involved in the regulation of Pol I machinery via the phosphorylation of Pol I and its transcription co-activators. EGF and serum stimulation may facilitate ERK-mediated phosphorylation of UBF and TIF-IA to enhance rRNA elongation and synthesis [38,39,40]. Serving as an important signaling pathway downstream of growth factors, AKT/mTOR may promote the transcriptional activity of Pol I via modulating the phosphorylation of TIF-IA [41,42]. Ribosome biogenesis is also coupled with cell-cycle progression via CDK4/CDK2-mediated UBF phosphorylation and activation [43]. Cumulatively, these studies suggested a tight regulation of rRNA transcription by mitogenic signals.

Metabolic status represents another important signal to modulate rRNA transcription. Ribosome biogenesis is an energy-intensive process and needs to be coordinated with an adequate energy status. Nutrient deprivation may cause rapid phosphorylation of TIF-IA at S635 residue by AMPK, leading to the inactivation of TIF-IA and the shutdown of rRNA transcription [44]. Likewise, mTOR-mediated TIF-IA phosphorylation at its N-terminal residues also couples nutrient availability to the adaptive increase of rRNA synthesis [27,45]. In addition to phosphorylation regulation by nutrient/energy-sensing kinases, protein acetylation is also critically involved in the coordination between energy availability and rRNA transcription. Multiple subunits of Pol I transcription machinery, including TIF-IB/SL1 and UBF, are regulated via acetylation modification by metabolic signaling pathways [46,47,48]. Moreover, rDNA availability is epigenetically regulated by sirtuins (Sirts) downstream of cellular energy status. It was revealed that energy status may activate a Sirt1-containing epigenetic complex termed energy-dependent nucleolar silencing complex (eNoSC), which in turn catalyzes the deacetylation and dimethylation of histone H3, resulting in rDNA silencing [49]. These data implicate a crucial role of metabolic signaling in the regulation of Pol I-mediated rRNA transcription.

In addition, rRNA transcription and ribosome biogenesis are controlled by stress signals and cell physiology. For instance, studies revealed that rDNA may serve as a sensor of genome integrity, and, when damaged, causes ATM-mediated inhibition of rRNA transcription [50]. This effect leads to aberrant nucleolar architecture and nucleolar protein shuttling, serving as a key event initiating nucleolar stress. JNK-mediated TIF-IA phosphorylation on T200 residue couples stress signals to the impairment in rRNA transcription [51]. Furthermore, nucleolar protein profiles are diverse among different cell lineages and associated with cell pluripotency and differentiation, which is elaborated in Section 5 [52].

## 4. The Role of the Nucleolus as a Signal Hub and Stress Sensor

**Figure 2:**
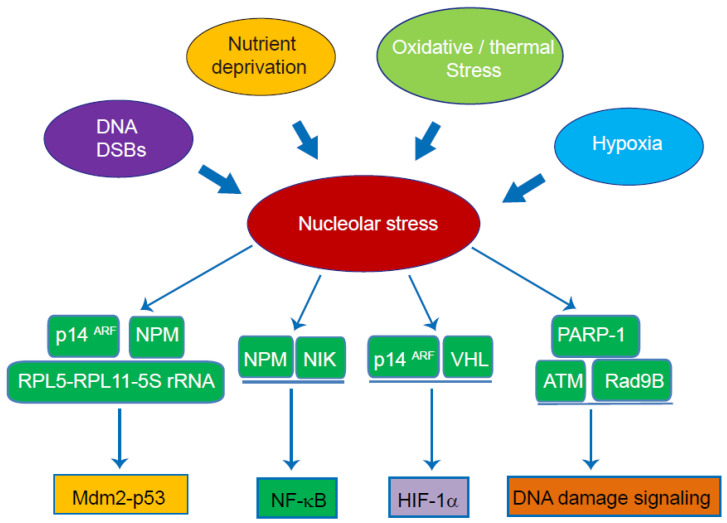
The role of nucleolar stress in transducing stress-related pathways. Nucleolar stress is initiated by various stress conditions, including DNA DSBs, nutrient deprivation, hypoxia, oxidative and thermal stress. Under these stress conditions, the nucleolus may modulate the transduction of various stress-associated pathways via aberrant nucleolar sequestration or release of different regulators.

As stated, nucleolar proteins may shuttle between the nucleolus and the nucleoplasm. Multiple nucleolar proteins, including NPM and nucleostemin (NS), shuttle continuously to coordinate the function of these proteins within and outside the nucleolus [53,54]. The initiation of nucleolar stress, as a result of rRNA transcription inhibition and nucleolar architecture disruption, may lead to aberrant shuttling of nucleolar proteins, causing their release from the nucleolus as well as nucleolar sequestration of various non-nucleolar proteins [14]. It has been revealed that nucleolar stress serves as a sensing hub of a wide range of stress stimuli, including DNA double strand breaks (DSBs), hypoxia, nutrient deprivation, oxidative and thermal stress [55,56]. Furthermore, nucleolar stress has been well-recognized to be an indispensable player in stress response via the initiation of various stress-related signaling pathways [57]. Studies in the past two decades have revealed a critical role of the nucleolus in the regulation of various signaling pathways, including the Mdm2-p53, NF-κB and HIF-1α pathways, in response to various stress conditions. Herein, we briefly discuss the mechanisms underlying nucleolus-initiated signaling pathways (Figure 2).

### 4.1. Mdm2-p53 Pathway

Mdm2-p53 signaling is a most well-documented stress response pathway downstream of nucleolar stress [58]. p53 has a rapid turnover rate due to Mdm2/MDMX-mediated ubiquitination and proteolysis in quiescent cells [59]. Early studies revealed that nucleolar disruption plays an indispensable role in p53 activation under DNA damage and other stress stimuli [60,61]. Currently, the detailed mechanisms underlying nucleolar stress-driven p53 activation remain under debate. Various hypotheses have been proposed to explain the causal role of nucleolar stress in p53 stabilization. One speculation is that nucleolar stress causes the redistribution of various ribosomal proteins into the nucleoplasm, where they interact with Mdm2 and MDMX. This interaction leads to the inactivation of Mdm2 and MDMX and subsequent p53 stabilization [62,63]. At present, a significant number of ribosomal proteins have been reportedly involved in the ribosomal protein-Mdm2-p53 pathway, and the list is still growing. Among them, RPL5 and RPL11 are of particular interest, because these two proteins, together with 5S rRNA, form the 5S ribonucleoprotein particle (RNP) complex to directly facilitate the Mdm2-p53 pathway [64,65]. In addition to ribosomal proteins, studies also revealed that the shuttling of p14^Arf^ may play a role in the Mdm2-p53 pathway. The release of p14^Arf^ from the nucleolus under nucleolar stress contributes to p53 activation [66,67]. Additionally, NPM may also physically bind to the Mdm2-p53 complex, triggering Mdm2 inactivation and p53 accumulation under stress conditions [54,68]. It is widely regarded that the Mdm2-p53 pathway plays a pivotal role in transducing nucleolar stress-mediated signaling and cell-fate decision, including apoptosis, senescence and growth arrest.

### 4.2. NF-κB Pathway

NF-κB transcription factors play a central role in immune regulation, stress response and maintaining cellular homeostasis [69]. The NF-κB family is composed of five different member proteins, including p50 (NF-kB1), p52 (NF-kB2), p65 (RelA), RelB and c-Rel [70]. While NF-κB signaling is mainly primed by extracellular cytokines, mounting studies have revealed that the NF-κB pathway may be initiated by a variety of intracellular stress signaling. An early study revealed a physical interaction between NPM and NF-κB proteins [71]. Their results also indicated the NPM function as a transcriptional co-activator of NF-κB in driving the expression of Manganese superoxide dismutase (MnSOD). Meanwhile, Andreas Birbach et al. [72] showed that NF-κB-inducing kinase (NIK) shuttles between the nucleolus and the nucleoplasm, and that nucleolar sequestration of NIK may reduce NF-κB activation potential. Of great intrigue, studies revealed that NF-κB proteins, particularly RelA, also display apparent nucleolar translocation. Lesley Stark et al. [73] reported that multiple pro-apoptotic stimuli, including aspirin, serum withdrawal and UV-C radiation, led to nucleolar sequestration of RelA and impaired NF-κB transcriptional activity. Nucleolar sequestration of RelA may trigger its ubiquitination and also cause aberrant localization of NPM during cell apoptosis [74,75]. On the other hand, both pharmacological and genetic induction of nucleolar stress, such as actinomycin D treatment and depletion of TIF-IA, may cause NF-κB nuclear translocation and activation [76,77,78]. Of note, treatment with a more selective nucleolar stress inducer CX-5461 did not cause NF-κB phosphorylation and activation, suggesting that NF-κB activation may occur independently of disruption of rRNA transcription [78,79].

### 4.3. HIF-1α

Serving as an important stress-responsive transcription factor, HIF-1α also displays nucleolar translocation. It was reported that p14^ARF^ may physically interact with HIF-1α and repress its transcriptional activity via nucleolar sequestration in a manner independent of p53 status [80]. Notably, VHL, the major E3 ligase catalyzing HIF-1α ubiquitination and degradation, undergoes nucleolar sequestration following hypoxia induction or normoxic acidosis [81]. The nucleolar sequestration of VHL is indispensable for the regulation of HIF-1α stability following returning hypoxia to normal oxygen, suggesting an integral role of the nucleolus in the regulation of HIF-1α activation under hypoxia. Moreover, nucleolar protein SENP3 (sentrin/SUMO-specific protease 3) may catalyze the de-SUMOylation of p300 to increase the transcriptional activity of HIF-1α, following ROS-induced release of SENP3 from nucleoli into nucleoplasm [82]. Moreover, given the fact that hypoxia and ROS may result in rRNA synthesis impairment and nucleolar condensation via acidosis-induced interaction between VHL and rDNA, it is speculated that the initiation of nucleolar stress may play a crucial role in the activation of HIF-1α under these conditions [83,84]. These findings suggest that the nucleolus may play a facilitating role in stress-induced HIF-1α activation. Much of this content remains incompletely understood and needs further investigations.

### 4.4. DNA Damage-Induced Stress Signalling

Various studies have indicated that the nucleolus may serve as a sensor to monitor genomic DNA stability and integrity. rDNA are among the most intensively transcribed chromatin regions in proliferating cells [85,86]. Due to robust rRNA transcription and its repetitive nature, rDNA is highly susceptible to DNA damages. DNA double-strand breaks (DSBs) and other forms of DNA damage may slow down or stall Pol I transcriptional machinery, which eventually leads to aberrant nucleolar dynamics and nucleolar stress [12]. Moreover, both functional and proteomic studies have revealed the apparent nucleolar localization of various DNA repair proteins [87]. For instance, 40% of total Poly-ADP ribose polymerase-1 (PARP1) is distributed within the nucleolus, and following DNA damage, this proportion of PARP1 is activated and released into the nucleoplasm [88]. In addition to PARP1, DSBs in the rDNA region lead to the recruitment of ataxia-telangiectasia-mutated (ATM) and ataxia-telangiectasia- and Rad3-related (ATR) into nucleolar caps, where they impair rRNA transcription and promote rDNA repair [89,90,91]. Rad9B, a component of the Rad9-Rad1-Hus1 (9-1-1) DNA checkpoint clamp, also migrates to the nucleolus following nucleolar stress and delay cell-cycle progression after DNA damage [92]. The interplay between DNA damage and nucleolar homeostasis is complicated and involves delicate regulatory mechanisms, which is not elaborated in this review (for more details, see [93,94]). Collectively, rDNA damage and nucleolar stress may play an integral role in DNA damage-initiated stress responses. Notably, many of these stress-response mechanisms function through p53-independent mechanisms [95,96].

### 4.5. Others

The nucleolar distribution of diverse proteins has been frequently observed, including cell-cycle regulators and metabolic and stress signaling transducers. Multiple cell-cycle proteins, such as cyclin E, CDK2, CDKN1A/p21, CDKN1B/p27, may display nucleolar distribution in diverse cell types [97,98,99,100]. Notably, nucleolar distribution may facilitate the ubiquitination of cell-cycle regulators, suggesting a crucial role of the nucleolus in cell-cycle regulation [100,101]. Likewise, c-Myc has also been reported to exhibit nucleolar accumulation and ubiquitination, leading to its proteolysis by proteasomes that are colocalized with c-Myc in the nucleolus [102,103]. The sirtuin proteins Sirt6 and Sirt7 also show significant nucleolar localization [104]. These NAD^+^-dependent deacetylases play a crucial role in the regulation of ribosome biogenesis, as well as metabolic control [105,106]. Nucleolar stress is also linked to Sirt1 expression and function, and its mechanism remains to be determined [107]. Moreover, the nucleolus may play a potential role in mitochondrial function and oxidative stress via modulating metabolic regulators [108]. Recent studies also revealed a critical role of the nucleolus in nuclear protein quality control [109,110,111]. Upon thermal stress, misfolded nuclear proteins are accumulated in the GC compartment of the nucleolus, where they are subjected to refolding or degradation. These findings implicate emerging roles of the nucleolus in stress responses and intracellular signaling regulation.

## 5. Nucleolus and Cell-Fate Decision

Because of the importance of the nucleolus and nucleolar stress in stress responses, it is conceivable that nucleolar physiology and stress participate in the decision of cell destiny [112]. Studies in the past decades have uncovered that nucleolar stress plays an integral role in cellular responses to various damaging stress conditions, and also participates critically in the modulation of cell fate during stress responses. Intriguingly, mounting investigations have revealed that nucleolar stress may trigger distinct behaviors in different cell lineages, suggesting complicated mechanisms by which the nucleolus participates in stress responses and cell-fate decision [113,114]. Additionally, the transformation of nucleolar morphology is essential for some physiological processes, such as stem cell differentiation, highlighting an indispensable role of the nucleolus and nucleolar stress in cell-fate decision.

### 5.1. Cell Division

As aforementioned, the coordinate regulation of ribosome biogenesis underlies mitogenic signal-driven cell division. While ribosome biogenesis is regulated downstream of growth signals, aberrant rRNA transcription may hamper cell proliferation in a feedback mechanism. The mechanisms underlying nucleolar stress-induced cell division arrest involve assorted signaling pathways. In this regard, both p53-dependent and -independent pathways play pivotal roles [115]. As one of the best-characterized stress pathways, p53 participates in a range of responses in response to nucleolar stress. A landmark study conducted by Rubbi CP et al. [60], revealed that blocking Pol I transcription alone, without the initiation of DNA damage, induces p53-dependent cell-cycle arrest. In addition, several groups showed that nucleolar stress may trigger cell-cycle arrest in p53-null cells. Giulio Donati et al. [116] reported that interference of Pol I subunit POLR1A resulted in the downregulation of the transcription factor E2F1 by the release of the ribosomal protein L11, leading to resultant cell-cycle arrest in p53-deficient cells. Another study showed that the blockade of ribosome biogenesis may destabilize serine/threonine kinase PIM1 in terms of inducing its proteasomal degradation, leading to subsequent p27 accumulation and cell-growth arrest in p53-null leukemia cells [117]. This data coincides with our report showing nucleolar localization and degradation of p27 in HCC [100]. Nucleolar stress may also regulate c-Myc expression to retard cell proliferation [118,119]. Taken together, these studies suggested an integral role of nucleolar stress in p53-dependent and -independent cell-cycle arrest.

### 5.2. Stemness and Differentiation

In addition to cell division, rRNA transcription and ribosome biogenesis are also involved in cell pluripotency [52]. Accumulating data suggested that nucleolar proteins and morphology differ between stem and differentiated cells [120,121]. Unlike tissue progenitors and somatic cells that possess multiple small nucleoli, embryonic stem cells (ESCs) contain 1–2 large nucleoli [122]. ESCs are also characterized by open chromatin conformation and high transcriptional activity in rDNA regions [123]. Mechanistic data revealed that the binding of pluripotency factors OCT4 and c-Myc underlies the hyperactive transcription of rDNA genes [124,125]. In contrast, differentiated cells exhibit comparably lower ribosome biogenesis and cluster of heterochromatin that compartments nucleoli into smaller ones [123]. Notably, this transition of nucleolar heterochromatin is essential for ESC differentiation and heterochromatinization in the ESC genome.

Multiple nucleolar proteins, such as NS, FBL and nucleolin (NCL), may be highly enriched in stem cells, and play indispensable roles in stem cell renewal [126,127,128]. The molecular mechanisms may involve the regulation of p14^ARF^ and p53. Recent investigations have revealed that epigenetic modifications, such as H3K4me3 and H3K27me3, are also involved in the maintenance of pluripotency [125,129]. Hui Zhang et al. [129] reported that DEAD-Box helicase 18 (DDX18) physically interacts and sequesters PRC2 in the out layer of the nucleolus to prevent PRC2-mediated H3K27me3 modification and transcriptional suppression at rDNA loci. In line with these studies, forced downregulation of rRNA transcription by treatment with actinomycin D or RNA interference of TIF-IA and FBL induces growth retardation and stem cell differentiation [128,130]. Collectively, these findings indicate that high rRNA transcription and nucleolar activity are hallmarks of cell stemness.

### 5.3. Apoptosis

Mounting studies have established a link between nucleolar stress and cell death induction. As indicated, nucleolar stress facilitates the p53 pathway and DDR-induced stress events. Nucleolar stress-induced apoptosis is critically involved in neuronal apoptosis in neurotoxic and neurodegenerative conditions [114,131]. The initiation of nucleolar stress participates in p53-dependent neuronal apoptosis following camptothecin (CPT)-induced DNA damage [114]. The involvement of nucleolar stress in apoptotic induction is also supported by genetic data. Xuejun Yuan el al [61] reported that the genetic ablation of TIF-IA causes nucleolar disappearance, cell-cycle arrest, p53 activation and apoptotic induction of cultured embryonic fibroblasts (MEFs). Selective inhibition of rRNA transcription by treatment with CX-5461 promotes caspase-3 activation and the apoptosis of multiple cell lineages [119,132,133]. Given the fact that nucleolar stress is initiated in a wide range of stress conditions, such as nutrient deprivation and genotoxic stress, and is critical to stress-related signaling, an involvement of nucleolar stress in the induction of apoptotic death under these stress conditions is speculated.

### 5.4. Senescence

Cell senescence is a state of permanent exit from the cell cycle accompanied with distinct morphological changes and secretary phenotypes [134]. Senescence may be initiated by various stress conditions such as replicative stress, oxidative damage and accumulation of DNA damages [135]. Compared with normal somatic cells, senescent cells display enlarged and fragmented nucleoli [136]. Recent studies have implicated an association between nucleolar stress and the induction of cell senescence [113,137]. Through the Cre/loxp-induced deletion of TIF-IA in mouse smooth muscle cells (MSCs), Wenjing Zhang et al. [113] reported that nucleolar stress causes a non-canonical DDR, p53 phosphorylation and senescent-phenotype in MSCs. Nucleolar stress is also involved in oncogene-induced senescence (OIS). Recent studies revealed that MYCN or H-Ras hyperactivation may initiate nucleolar stress to trigger the p53-p21 pathway, cell-cycle arrest and senescence-like characteristics in different cell lineages [138,139]. Likewise, the pharmacological inhibition of rRNA transcription elicits a senescence-promoting effect in multiple cancer cell lines [140,141]. Notably, most of these studies pointed to a pivotal role of the p53 pathway in nucleolar stress-induced senescence phenotypes.

### 5.5. Autophagy

Autophagy represents a critical stress event in response to various unfavorable conditions including nutrient deprivation, hypoxia and oxidative and thermal stresses [142]. These stress conditions may inactivate mTOR signaling and activate catabolic energy-generating pathways such as AMPK, leading to the activation of ULK kinases and the subsequent phosphorylation of autophagy-associated gene 13 (ATG13) and FIP200. The assembly of ULK-ATG13-FIP200 complex causes the recruitment of other ATG proteins, which in turn leads to the formation of autophagosome, and as a result, the initiation of autophagy [143,144]. Autophagy serves as a highly conserved mechanism to degradate and recycle cytoplasmic organelles and macromolecules, to handle adverse environments. Studies revealed that nucleolar stress may cause the initiation of the autophagic program. Nucleolar stress has been reportedly involved in the regulation of autophagy-associated pathways, such as mTOR [108]. Inhibition of Pol I transcription both using pharmacological and genetic approaches leads to the initiation of autophagy in a manner independent of p53 [145]. More recent data have revealed that nucleolar stress may trigger autophagy through upregulating ATG proteins [146]. Notably, multiple studies confirmed that treatment with the selective inhibitor of rRNA transcription CX-5461 resulted in apparent autophagy in various cancer cell types [141,147,148]. The induction of autophagy may be attributed to the alteration of mTOR and AMPK pathways, and may also involve the p53 pathway [147]. It was revealed that depletion of p53 attenuated autophagy and cell death following CX-5461 treatment. However, the underlying mechanisms remain incompletely understood and need further investigations.

## 6. Nucleolus, Nucleolar Stress and Disease Development

Because of its importance in stress responses and signaling transduction, the nucleolus has been conceivably involved in the development of assorted human diseases. Given the fact that nucleolar stress is initiated in various unfavorable stress conditions and participates in stress-induced cell-fate decision, its involvement in tumorigenesis, neurodegenerative diseases and metabolic disorders has been increasingly recognized [149]. Studies revealed that nucleolar stress is involved in cell damage and death under a variety of pathological conditions, particularly in neuronal death during neurodegeneration [150]. On the other hand, robust nucleolar activity has been widely regarded as a hallmark and therapeutic vulnerability of assorted types of carcinomas. Due to the significant differences of nucleolar morphology and activity between normal and malignant cells, nucleolar stress as a tumor-selective stress event has attracted significant therapeutic attention [151]. These discoveries implicate a promising future of nucleolar intervention in the treatment of these diseases.

### 6.1. Cancer Development and Therapy

The hyperactivation of nucleolar rRNA synthesis has been well-documented to fuel malignant behaviors of tumor cells. In addition to an essential role in ribosome biogenesis, the cancerous nucleolus also plays an integral role in transmitting oncogenic signaling. NPM overexpression is associated with worsened prognosis in solid tumors [152]. Some nucleolar proteins, such as NS and Sirt7, also exhibit oncogenic properties via molecular mechanisms independent of ribosome biogenesis [153,154,155]. Multiple signals discussed in Section 4, such as NF-κB and HIF-1α, play central roles in tumor initiation and progression, suggesting that the nucleolus may constitute an oncogenic hub for tumor aggressiveness.

Of great intrigue, because of an active role in tumorigenesis, the nucleolus has been widely viewed as a therapeutic vulnerability for cancers [12]. The blockade of rRNA transcription may selectively cause stress and damage to tumor cells. Megan Bywater et al. [156] reported that the inhibition of rRNA transcription using CX-5461 may selectively kill B-lymphoma cells, but spare normal B cells, in an Eμ-Myc lymphoma xenograft model. Their study uncovered a tumor-specific activation of p53-dependent apoptotic signaling following the induction of nucleolar stress using CX-5461. In addition to the p53 pathway, nucleolar stress also drives the nucleolar release of ribosomal protein uL3, which in turn interacts with PARP-1 and suppresses PARP-1-dependent activation of E2F1 and cyclin D1 expression, eventually leading to cell-cycle arrest [157]. Notably, these effects of nucleoplasmic uL3 are not compromised in p53-null cells. These data together implied that nucleolar stress may selectively cause viability impairment and growth arrest via both p53-dependent and –independent mechanisms.

The development of a highly selective inhibitor of Pol I, CX-5461, has significantly accelerated the implication of targeting the nucleolus in cancer treatment [141,158]. Since its discovery, mounting pre-clinical studies have indicated its potent anti-tumor effects both in vitro and in vivo [133,159]. As indicated by previous studies, the effects of CX-5461 involve various stress responses in tumor cells, including cell-cycle arrest, apoptosis, autophagy and senescence, all of which, to some extent, constitute anti-tumor effects of CX-5461 [119,141]. Moreover, recent outcomes of clinical trials have also indicated a promising potential for clinical use in hematologic and solid tumors [160,161]. These clinical trials have demonstrated that CX-5461 is generally well-tolerant and may induce partial tumor remission in patients with late-stage cancers. With the release of more clinical trial results in the coming years, better knowledge of the therapeutic merit of CX-5461 is anticipated.

### 6.2. Neurodegenerative Diseases

An association between nucleolar stress and neurodegeneration has been observed since more than one decade ago [108]. Currently, nucleolar stress has been linked to the pathogenesis of various neurodegenerative diseases, such as Alzheimer’s disease (AD), Parkinson’s disease (PD), Huntington’s disease (HTT) and amyotrophic lateral sclerosis (ALS) [150,162,163]. rDNA hypermethylation and transcriptional silencing have been observed in the cerebrocortical tissues of subjects with early- and late-stage AD [164]. Likewise, disruption of nucleolar integrity has been observed in postmortem brain specimens of individuals with PD [108]. An experimental PD model induced by neurotoxin MPTP also recaptured nucleolar disruption and impaired rRNA synthesis, which in turn is associated with oxidative stress, p53 activation and impaired mTOR signaling. Mechanistic insights indicated that α-synuclein and DJ-1, two important proteins involved in PD development, interact with NCL and may alter rRNA synthesis [165,166]. Aberrant acetylation of TIF-IA and impaired rRNA transcription were also reportedly involved in the pathogenesis of HTT [167]. More recent studies revealed the integral roles of nucleolar disruption and aberrant rRNA processing in the development of HTT [168]. Collectively, these findings implicate the damage of nucleolar architecture and rRNA synthesis serving as a stress hub and pro-death signal during the pathogenesis of various neurodegenerative diseases.

The disruption of the nucleolar function may be attributed to the direct accumulation of protein aggresomes in the neuronal nucleolus [169]. Nucleolar accumulation of pathological factors, such as misfold neurotoxic proteins and RNAs, has been frequently observed in various neurodegenerative conditions [131,170,171]. Their mislocalization in the nucleolus may suppress ribosome biogenesis by directly binding to and inactivating proteins critical for rRNA transcription [150,172]. As a consequence, impaired rRNA synthesis resulted in the alternation of nucleolar architecture and the activation of downstream stress response pathways, such as Mdm2-p53 signaling, eventually leading to neuronal damage and apoptosis [150]. The disruption of nucleolar integrity is also manifested by the mislocalization and altered expression of nucleolar proteins [165,173]. Delocalization of NPM and NCL in the nucleolus was observed in various models of neurodegenerative diseases [168,174]. Of note, studies also indicated that the overexpression of NPM and NCL may elicit neuroprotective roles in in vitro culture models [165,173]. Taken together, these data demonstrate an integral role of nucleolar disruption in the development of neurodegenerative diseases and implicate that the protective intervention of the nucleolus may prevent the progression of neurodegenerative diseases.

### 6.3. Metabolic Diseases

While the direct evidence linking nucleolar stress to metabolic disorders remains scarce, an influence of nutrient availability on nucleolar activity has been well-established. Nutrient-sensing pathways, such as AMPK and mTOR, have been implicated in the regulation of rRNA synthesis and nucleolar homeostasis [27,44]. Intriguingly, studies indicated a role of snoRNAs in mediating metabolic stress and lipotoxicity [175,176]. snoRNAs are small, noncoding RNAs mainly localized in the nucleolus and play indispensable roles in rRNA chemical modifications and processing [177]. snoRNAs are composed of two major classes, the box C/D and the box H/ACA snoRNAs, with a nucleotide length of 60–90 bp and 120–140 bp, respectively. Apart from its role in ribosome biogenesis, accumulating evidence indicates a noncanonical function of snoRNAs in alternative splicing of various pre-mRNAs [178]. Moreover, the expression and distribution of snoRNAs are regulated by intracellular signaling and stress conditions [179,180]. Carlos Michel et al. [175] reported that multiple snoRNAs encoded in the ribosomal protein L13a (Rpl13a) locus are induced and elicit a pro-apoptotic effect following exposure to saturated fatty acids. Their lab later revealed that ablation of these snoRNAs alters mitochondrial function and impairs the generation of reactive oxygen species (ROS), which in turn improves glucose-stimulated insulin secretion and systemic glucose tolerance in mice [181]. While the link between nucleolar stress and the function of these snoRNAs remains incompletely understood, these data implicated an involvement of nucleolar RNA in sensing and regulating metabolic stress.

A study obtained from C. elegans suggested that nucleolar stress triggered by ablation of multiple proteins involved in ribosome biogenesis initiates lipid accumulation via facilitating the PHA-4/FoxA-mediated expression of lipogenic genes [182]. This finding implicates that nucleolar stress may directly regulate lipid metabolism, at least in invertebrates. Moreover, data from the mammalian system also confirmed a key role of the nucleolus in metabolic regulation. Ablation of Sirt7 has been reported to alter hepatic lipid metabolism and the development of fatty liver diseases, albeit inconsistent results were obtained by different groups [106,183,184,185]. Sirt7 is mainly distributed in the nucleolus in quiescent cells, and upon nucleolar stress, is released into the nucleoplasm and the cytoplasm, where it may alter the acetylation of metabolic regulators [185,186]. Another study by genetically disrupting ribosome protein (RP)-Mdm2 binding revealed a crucial role of RP-Mdm2-p53 pathway in lipid metabolism under nutrient stress [187]. Mice with disrupted interaction between RP and Mdm2 developed apparent hepatosteatosis under acute fasting conditions [187]. Mechanistic data indicated that nucleolar stress induced by nutrient deprivation caused the p53-mediated transactivation of malonyl-CoA decarboxylase (MCD) and lipid accumulation in the liver. Combined, these data indicated that the nucleolus is a key player in nutrient metabolism and the development of metabolic disorders.

### 6.4. Aging and Longevity

Some early observations have revealed that the nucleolus plays a key role in the regulation of the longevity of yeast and C. elegans [188,189]. One study showed that the nucleolar localization of longevity-associated protein Sir4 may increase the life span of yeast [189]. Coinciding with the notion that the nucleolus is involved in the regulation of life span, more recent studies have revealed that reduced rRNA transcription and nucleolar size are correlated with the longevity of C. elegans [190]. On the contrary, increased rRNA production and enlarged nucleoli are hallmarks of the premature aging of fibroblasts derived from Hutchinson–Gilford progeria syndrome (HGPS) patients [191]. In this study, authors also observed smaller nucleoli in muscle biopsies of human subjects undergoing calorie restriction. Given the fact that nucleolar stress triggers cellular senescence, small nucleoli and reduced rRNA synthesis may confer resistance to nucleolar stress in these cells.

Notably, mechanistic dissection uncovered that rDNA hypermethylation is positively correlated with the aging of human and rat hepatocytes [192]. This link is also validated by the fact that calorie restriction may reverse the methylation of rDNA and marginally reduce rRNA synthesis [192,193]. In line with these findings, epigenetic landscape data confirmed that rDNA CpG methylation is strongly correlated with the aging of diverse organisms, including yeast, C. elegans and human [194]. rDNA methylation may precisely predict the aging status of different individual within species. In addition, the intervention of the life span using environmental and genetic approaches alters rDNA methylation, suggesting a coordination between rDNA methylation and cell aging [194]. These findings indicate that aging cells exhibit both enhanced rRNA transcription and rDNA hypermethylation. Hence, a conflict between the reduced general transcriptional capacity of rDNA caused by rDNA methylation and higher rRNA transcription may result in the accumulation of rDNA damage and nucleolar defects, eventually leading to aging program [195]. Overall, these findings demonstrated an indispensable role of the nucleolus and rDNA activity in the regulation of aging and longevity.

## 7. Conclusions and Future Direction

Though a central role of the nucleolus in ribosome biogenesis has been revealed since half a century ago, it was only until recent years that the involvement of the nucleolus in stress response and disease progression has attracted significant research attention [196]. To date, the role of the nucleolus in stress response has been well-recognized, and its implication in the development of various human diseases has increasingly become a focus of study. However, much of this field remains incompletely clarified and requires better understanding. In addition, much of our knowledge derives from some model organisms distinct from mammals, such as C. elegans and yeast. Thus, further investigations into the mechanisms underpinning its contributions to disease development are in urgent need.

The discovery of the nucleolus in the transduction of stress-responsive signals led to the concept of nucleolar stress more than a decade ago [13]. Currently, nucleolar stress has been associated with the development of a growing number of diseases. While its connection with some diseases, such as cancers, has been intensively investigated since decades ago, mechanistic insights into the roles of the nucleolus and nucleolar stress in many others have only emerged very recently [8,197]. Other than the diseases listed in Section 5, recent studies have established a link between nucleolar stress and the development of cardiovascular diseases, smooth muscle degeneration, innate immune responses and developmental disorders [113,198,199,200]. Even though the underlying mechanisms remain obscure, an involvement of nucleolar stress will undoubtedly provide novel insights into the mechanisms underlying the development of these diseases.

Ribosome biogenesis is an energy-intense process fundamental to cellular homeostasis. The coordination between ribosome biogenesis and homeostatic status plays a critical role in the transition of cell physiology [22,201]. While nucleolar stress initiated by the acute disruption of ribosome biogenesis may lead to stress-associated changes of cell physiology such as apoptosis, senescence and autophagy, nucleolar stress itself plays both protective and detrimental roles in cell physiology and disease progression [202,203]. In this regard, multiple nucleolar proteins, such as NPM, NS and NCL, elicit a pro-survival and -repair function, especially during the early stages of chronic diseases [204,205]. Forced expression of these proteins has been shown to protect somatic cells from damage and death during various disease models [173,204,206]. Thus, despite the fact that the nucleolus participates critically in the development of assorted human diseases, the initiation of nucleolar stress may, to some extent, prevent the deterioration of tissue injury and degeneration. Intervening strategies that selectively activate the pro-survival and -repair function of the nucleolus may be promising approaches to prevent the development of tissue degeneration and failure.

In summary, the role of the nucleolus in stress sensing, cell-fate decision and disease progression has increasingly become a focus of study. A deeper understanding of the underlying molecular mechanisms will shed new light on the pathogenesis of various human diseases.

## Data Availability

Not applicable.

## References

[1] Penzo M., Montanaro L., Treré D., Derenzini M. (2019). The Ribosome Biogenesis—Cancer Connection. Cells.

[2] Sakai K., Ohta T., Minoshima S., Kudoh J., Wang Y., de Jong P.J., Shimizu N. (1995). Human ribosomal RNA gene cluster: Identification of the proximal end containing a novel tandem repeat sequence. Genomics.

[3] Lafontaine D.L.J., Riback J.A., Bascetin R., Brangwynne C.P. (2021). The nucleolus as a multiphase liquid condensate. Nat. Rev. Mol. Cell Biol..

[4] Puvion-Dutilleul F., Puvion E., Bachellerie J.P. (1997). Early stages of pre-rRNA formation within the nucleolar ultrastructure of mouse cells studied by in situ hybridization with a 5′ETS leader probe. Chromosoma.

[5] Schmidt E.V. (1999). The role of c-myc in cellular growth control. Oncogene.

[6] Mahoney S.J., Dempsey J.M., Blenis J. (2009). Cell signaling in protein synthesis ribosome biogenesis and translation initiation and elongation. Prog. Mol. Biol. Transl. Sci..

[7] Németh A., Grummt I. (2018). Dynamic regulation of nucleolar architecture. Curr. Opin. Cell Biol..

[8] Derenzini M., Trere D., Pession A., Montanaro L., Sirri V., Ochs R.L. (1998). Nucleolar function and size in cancer cells. Am. J. Pathol..

[9] Pich A., Chiusa L., Margaria E. (2000). Prognostic relevance of AgNORs in tumor pathology. Micron.

[10] Piffko J., Bankfalvi A., Ofner D., Bryne M., Rasch D., Joos U., Bocker W., Schmid K.W. (1997). Prognostic value of histobiological factors (malignancy grading and AgNOR content) assessed at the invasive tumour front of oral squamous cell carcinomas. Br. J. Cancer.

[11] Bahadori M., Azizi M.H., Dabiri S. (2018). Recent Advances on Nucleolar Functions in Health and Disease. Arch. Iran. Med..

[12] Weeks S.E., Metge B.J., Samant R.S. (2019). The nucleolus: A central response hub for the stressors that drive cancer progression. Cell Mol. Life Sci..

[13] Mayer C., Grummt I. (2005). Cellular stress and nucleolar function. Cell Cycle.

[14] Boulon S., Westman B.J., Hutten S., Boisvert F.M., Lamond A.I. (2010). The nucleolus under stress. Mol. Cell.

[15] Biggiogera M., Martin T.E., Gordon J., Amalric F., Fakan S. (1994). Physiologically inactive nucleoli contain nucleoplasmic ribonucleoproteins: Immunoelectron microscopy of mouse spermatids and early embryos. Exp. Cell Res..

[16] Engel W., Zenzes M.T., Schmid M. (1977). Activation of mouse ribosomal RNA genes at the 2-cell stage. Hum. Genet..

[17] Musinova Y.R., Kananykhina E.Y., Potashnikova D.M., Lisitsyna O.M., Sheval E.V. (2015). A charge-dependent mechanism is responsible for the dynamic accumulation of proteins inside nucleoli. Biochim. Biophys. Acta.

[18] Falahati H., Pelham-Webb B., Blythe S., Wieschaus E. (2016). Nucleation by rRNA Dictates the Precision of Nucleolus Assembly. Curr. Biol..

[19] Riback J.A., Zhu L., Ferrolino M.C., Tolbert M., Mitrea D.M., Sanders D.W., Wei M.T., Kriwacki R.W., Brangwynne C.P. (2020). Composition-dependent thermodynamics of intracellular phase separation. Nature.

[20] (2017). Correction to Supporting Information for Falahati and Wieschaus, Independent active and thermodynamic processes govern the nucleolus assembly in vivo. Proc. Natl. Acad. Sci. USA.

[21] Maggi L.B., Kuchenruether M., Dadey D.Y., Schwope R.M., Grisendi S., Townsend R.R., Pandolfi P.P., Weber J.D. (2008). Nucleophosmin serves as a rate-limiting nuclear export chaperone for the Mammalian ribosome. Mol. Cell Biol..

[22] Ni C., Buszczak M. (2022). The homeostatic regulation of ribosome biogenesis. Semin. Cell Dev. Biol..

[23] Hempel W.M., Cavanaugh A.H., Hannan R.D., Taylor L., Rothblum L.I. (1996). The species-specific RNA polymerase I transcription factor SL-1 binds to upstream binding factor. Mol. Cell. Biol..

[24] Sanij E., Hannan R.D. (2009). The role of UBF in regulating the structure and dynamics of transcriptionally active rDNA chromatin. Epigenetics.

[25] Goodfellow S.J., Zomerdijk J.C. (2013). Basic mechanisms in RNA polymerase I transcription of the ribosomal RNA genes. Subcell. Biochem..

[26] Ford E., Voit R., Liszt G., Magin C., Grummt I., Guarente L. (2006). Mammalian Sir2 homolog SIRT7 is an activator of RNA polymerase I transcription. Genes Dev..

[27] Mayer C., Zhao J., Yuan X., Grummt I. (2004). mTOR-dependent activation of the transcription factor TIF-IA links rRNA synthesis to nutrient availability. Genes Dev..

[28] Fath S., Milkereit P., Peyroche G., Riva M., Carles C., Tschochner H. (2001). Differential roles of phosphorylation in the formation of transcriptional active RNA polymerase I. Proc. Natl. Acad. Sci. USA.

[29] Filipowicz W., Pelczar P., Pogacic V., Dragon F. (1999). Structure and biogenesis of small nucleolar RNAs acting as guides for ribosomal RNA modification. Acta Biochim. Pol..

[30] Yoshikawa H., Komatsu W., Hayano T., Miura Y., Homma K., Izumikawa K., Ishikawa H., Miyazawa N., Tachikawa H., Yamauchi Y. (2011). Splicing factor 2-associated protein p32 participates in ribosome biogenesis by regulating the binding of Nop52 and fibrillarin to preribosome particles. Mol. Cell. Proteom..

[31] Rodgers M.L., Woodson S.A. (2021). A roadmap for rRNA folding and assembly during transcription. Trends Biochem. Sci..

[32] Zhang Q., Shalaby N.A., Buszczak M. (2014). Changes in rRNA Transcription Influence Proliferation and Cell Fate Within a Stem Cell Lineage. Science.

[33] Larson D.E., Xie W., Glibetic M., O’Mahony D., Sells B.H., Rothblum L.I. (1993). Coordinated decreases in rRNA gene transcription factors and rRNA synthesis during muscle cell differentiation. Proc. Natl. Acad. Sci. USA.

[34] Lempiainen H., Shore D. (2009). Growth control and ribosome biogenesis. Curr. Opin. Cell Biol..

[35] Brown I.N., Lafita-Navarro M.C., Conacci-Sorrell M. (2022). Regulation of Nucleolar Activity by MYC. Cells.

[36] Arabi A., Wu S., Ridderstrale K., Bierhoff H., Shiue C., Fatyol K., Fahlen S., Hydbring P., Soderberg O., Grummt I. (2005). c-Myc associates with ribosomal DNA and activates RNA polymerase I transcription. Nat. Cell Biol..

[37] Grandori C., Gomez-Roman N., Felton-Edkins Z.A., Ngouenet C., Galloway D.A., Eisenman R.N., White R.J. (2005). c-Myc binds to human ribosomal DNA and stimulates transcription of rRNA genes by RNA polymerase I. Nat. Cell Biol..

[38] Stefanovsky V., Langlois F., Gagnon-Kugler T., Rothblum L.I., Moss T. (2006). Growth factor signaling regulates elongation of RNA polymerase I transcription in mammals via UBF phosphorylation and r-chromatin remodeling. Mol. Cell.

[39] Zhao J., Yuan X., Frodin M., Grummt I. (2003). ERK-dependent phosphorylation of the transcription initiation factor TIF-IA is required for RNA polymerase I transcription and cell growth. Mol. Cell.

[40] Stefanovsky V.Y., Langlois F., Bazett-Jones D., Pelletier G., Moss T. (2006). ERK modulates DNA bending and enhancesome structure by phosphorylating HMG1-boxes 1 and 2 of the RNA polymerase I transcription factor UBF. Biochemistry.

[41] Nguyen L.X.T., Mitchell B.S. (2013). Akt activation enhances ribosomal RNA synthesis through casein kinase II and TIF-IA. Proc. Natl. Acad. Sci. USA.

[42] Wu C., You J., Fu J., Wang X., Zhang Y. (2016). Phosphatidylinositol 3-Kinase/Akt Mediates Integrin Signaling To Control RNA Polymerase I Transcriptional Activity. Mol. Cell Biol..

[43] Voit R., Hoffmann M., Grummt I. (1999). Phosphorylation by G1-specific cdk-cyclin complexes activates the nucleolar transcription factor UBF. EMBO J..

[44] Hoppe S., Bierhoff H., Cado I., Weber A., Tiebe M., Grummt I., Voit R. (2009). AMP-activated protein kinase adapts rRNA synthesis to cellular energy supply. Proc. Natl. Acad. Sci. USA.

[45] James M.J., Zomerdijk J.C. (2004). Phosphatidylinositol 3-kinase and mTOR signaling pathways regulate RNA polymerase I transcription in response to IGF-1 and nutrients. J. Biol. Chem..

[46] Muth V., Nadaud S., Grummt I., Voit R. (2001). Acetylation of TAF(I)68, a subunit of TIF-IB/SL1, activates RNA polymerase I transcription. EMBO J..

[47] Pelletier G., Stefanovsky V.Y., Faubladier M., Hirschler-Laszkiewicz I., Savard J., Rothblum L.I., Cote J., Moss T. (2000). Competitive recruitment of CBP and Rb-HDAC regulates UBF acetylation and ribosomal transcription. Mol. Cell.

[48] Houston R., Sekine S., Calderon M.J., Seifuddin F., Wang G., Kawagishi H., Malide D.A., Li Y., Gucek M., Pirooznia M. (2020). Acetylation-mediated remodeling of the nucleolus regulates cellular acetyl-CoA responses. PLoS Biol..

[49] Murayama A., Ohmori K., Fujimura A., Minami H., Yasuzawa-Tanaka K., Kuroda T., Oie S., Daitoku H., Okuwaki M., Nagata K. (2008). Epigenetic control of rDNA loci in response to intracellular energy status. Cell.

[50] Kruhlak M., Crouch E.E., Orlov M., Montano C., Gorski S.A., Nussenzweig A., Misteli T., Phair R.D., Casellas R. (2007). The ATM repair pathway inhibits RNA polymerase I transcription in response to chromosome breaks. Nature.

[51] Mayer C., Bierhoff H., Grummt I. (2005). The nucleolus as a stress sensor: JNK2 inactivates the transcription factor TIF-IA and down-regulates rRNA synthesis. Genes Dev..

[52] Brombin A., Joly J.S., Jamen F. (2015). New tricks for an old dog: Ribosome biogenesis contributes to stem cell homeostasis. Curr. Opin. Genet. Dev..

[53] Meng L., Zhu Q., Tsai R.Y. (2007). Nucleolar trafficking of nucleostemin family proteins: Common versus protein-specific mechanisms. Mol. Cell Biol..

[54] Colombo E., Marine J.C., Danovi D., Falini B., Pelicci P.G. (2002). Nucleophosmin regulates the stability and transcriptional activity of p53. Nat. Cell Biol..

[55] Korsholm L.M., Gal Z., Nieto B., Quevedo O., Boukoura S., Lund C.C., Larsen D.H. (2020). Recent advances in the nucleolar responses to DNA double-strand breaks. Nucleic Acids Res..

[56] Gorner W., Durchschlag E., Wolf J., Brown E.L., Ammerer G., Ruis H., Schuller C. (2002). Acute glucose starvation activates the nuclear localization signal of a stress-specific yeast transcription factor. EMBO J..

[57] Yang K., Yang J., Yi J. (2018). Nucleolar Stress: Hallmarks, sensing mechanism and diseases. Cell Stress.

[58] Lindstrom M.S., Bartek J., Maya-Mendoza A. (2022). p53 at the crossroad of DNA replication and ribosome biogenesis stress pathways. Cell Death Differ..

[59] Shadfan M., Lopez-Pajares V., Yuan Z.M. (2012). MDM2 and MDMX: Alone and together in regulation of p53. Transl. Cancer Res..

[60] Rubbi C.P., Milner J. (2003). Disruption of the nucleolus mediates stabilization of p53 in response to DNA damage and other stresses. EMBO J..

[61] Yuan X., Zhou Y., Casanova E., Chai M., Kiss E., Grone H.J., Schutz G., Grummt I. (2005). Genetic inactivation of the transcription factor TIF-IA leads to nucleolar disruption, cell cycle arrest, and p53-mediated apoptosis. Mol. Cell.

[62] Gilkes D.M., Chen L., Chen J. (2006). MDMX regulation of p53 response to ribosomal stress. EMBO J..

[63] Daftuar L., Zhu Y., Jacq X., Prives C. (2013). Ribosomal proteins RPL37, RPS15 and RPS20 regulate the Mdm2-p53-MdmX network. PLoS ONE.

[64] Sloan K.E., Bohnsack M.T., Watkins N.J. (2013). The 5S RNP couples p53 homeostasis to ribosome biogenesis and nucleolar stress. Cell Rep..

[65] Donati G., Peddigari S., Mercer C.A., Thomas G. (2013). 5S ribosomal RNA is an essential component of a nascent ribosomal precursor complex that regulates the Hdm2-p53 checkpoint. Cell Rep..

[66] Llanos S., Clark P.A., Rowe J., Peters G. (2001). Stabilization of p53 by p14ARF without relocation of MDM2 to the nucleolus. Nat. Cell Biol..

[67] Lee C., Smith B.A., Bandyopadhyay K., Gjerset R.A. (2005). DNA damage disrupts the p14ARF-B23(nucleophosmin) interaction and triggers a transient subnuclear redistribution of p14ARF. Cancer Res..

[68] Kurki S., Peltonen K., Latonen L., Kiviharju T.M., Ojala P.M., Meek D., Laiho M. (2004). Nucleolar protein NPM interacts with HDM2 and protects tumor suppressor protein p53 from HDM2-mediated degradation. Cancer Cell.

[69] Chen J., Stark L.A. (2018). Crosstalk between NF-kappaB and Nucleoli in the Regulation of Cellular Homeostasis. Cells.

[70] Verma I.M. (2004). Nuclear factor (NF)-kappaB proteins: Therapeutic targets. Ann. Rheum. Dis..

[71] Dhar S.K., Lynn B.C., Daosukho C., St Clair D.K. (2004). Identification of nucleophosmin as an NF-kappaB co-activator for the induction of the human SOD2 gene. J. Biol. Chem..

[72] Birbach A., Bailey S.T., Ghosh S., Schmid J.A. (2004). Cytosolic, nuclear and nucleolar localization signals determine subcellular distribution and activity of the NF-kappaB inducing kinase NIK. J. Cell Sci..

[73] Stark L.A., Dunlop M.G. (2005). Nucleolar sequestration of RelA (p65) regulates NF-kappaB-driven transcription and apoptosis. Mol. Cell Biol..

[74] Thoms H.C., Loveridge C.J., Simpson J., Clipson A., Reinhardt K., Dunlop M.G., Stark L.A. (2010). Nucleolar targeting of RelA(p65) is regulated by COMMD1-dependent ubiquitination. Cancer Res..

[75] Khandelwal N., Simpson J., Taylor G., Rafique S., Whitehouse A., Hiscox J., Stark L.A. (2011). Nucleolar NF-kappaB/RelA mediates apoptosis by causing cytoplasmic relocalization of nucleophosmin. Cell Death Differ..

[76] Michel J., Nolin F., Wortham L., Lalun N., Tchelidze P., Banchet V., Terryn C., Ploton D. (2019). Various Nucleolar Stress Inducers Result in Highly Distinct Changes in Water, Dry Mass and Elemental Content in Cancerous Cell Compartments: Investigation Using a Nano-Analytical Approach. Nanotheranostics.

[77] Liu X.F., Xiang L., Zhou Q., Carralot J.P., Prunotto M., Niederfellner G., Pastan I. (2016). Actinomycin D enhances killing of cancer cells by immunotoxin RG7787 through activation of the extrinsic pathway of apoptosis. Proc. Natl. Acad. Sci. USA.

[78] Chen J., Lobb I.T., Morin P., Novo S.M., Simpson J., Kennerknecht K., von Kriegsheim A., Batchelor E.E., Oakley F., Stark L.A. (2018). Identification of a novel TIF-IA-NF-kappaB nucleolar stress response pathway. Nucleic Acids Res..

[79] Pan G., Zhang J., Han Y., Chen Y., Guo X., Cui X., Cheng M., Gao H., Wang J., Jiang F. (2022). CX-5461 is a potent immunosuppressant which inhibits T cell-mediated alloimmunity via p53-DUSP5. Pharmacol. Res..

[80] Fatyol K., Szalay A.A. (2001). The p14ARF tumor suppressor protein facilitates nucleolar sequestration of hypoxia-inducible factor-1alpha (HIF-1alpha) and inhibits HIF-1-mediated transcription. J. Biol. Chem..

[81] Mekhail K., Gunaratnam L., Bonicalzi M.E., Lee S. (2004). HIF activation by pH-dependent nucleolar sequestration of VHL. Nat. Cell Biol..

[82] Huang C., Han Y., Wang Y., Sun X., Yan S., Yeh E.T., Chen Y., Cang H., Li H., Shi G. (2009). SENP3 is responsible for HIF-1 transactivation under mild oxidative stress via p300 de-SUMOylation. EMBO J..

[83] Mekhail K., Rivero-Lopez L., Khacho M., Lee S. (2006). Restriction of rRNA synthesis by VHL maintains energy equilibrium under hypoxia. Cell Cycle.

[84] Yang K., Wang M., Zhao Y., Sun X., Yang Y., Li X., Zhou A., Chu H., Zhou H., Xu J. (2016). A redox mechanism underlying nucleolar stress sensing by nucleophosmin. Nat. Commun..

[85] Smirnov E., Chmurciakova N., Cmarko D. (2021). Human rDNA and Cancer. Cells.

[86] Kobayashi T. (2011). Regulation of ribosomal RNA gene copy number and its role in modulating genome integrity and evolutionary adaptability in yeast. Cell Mol. Life Sci..

[87] Ogawa L.M., Baserga S.J. (2017). Crosstalk between the nucleolus and the DNA damage response. Mol. Biosyst..

[88] Rancourt A., Satoh M.S. (2009). Delocalization of nucleolar poly(ADP-ribose) polymerase-1 to the nucleoplasm and its novel link to cellular sensitivity to DNA damage. DNA Repair.

[89] Harding S.M., Boiarsky J.A., Greenberg R.A. (2015). ATM Dependent Silencing Links Nucleolar Chromatin Reorganization to DNA Damage Recognition. Cell Rep..

[90] van Sluis M., McStay B. (2015). A localized nucleolar DNA damage response facilitates recruitment of the homology-directed repair machinery independent of cell cycle stage. Genes Dev..

[91] Korsholm L.M., Gal Z., Lin L., Quevedo O., Ahmad D.A., Dulina E., Luo Y., Bartek J., Larsen D.H. (2019). Double-strand breaks in ribosomal RNA genes activate a distinct signaling and chromatin response to facilitate nucleolar restructuring and repair. Nucleic Acids Res..

[92] Perez-Castro A.J., Freire R. (2012). Rad9B responds to nucleolar stress through ATR and JNK signalling, and delays the G1-S transition. J. Cell Sci..

[93] Lindstrom M.S., Jurada D., Bursac S., Orsolic I., Bartek J., Volarevic S. (2018). Nucleolus as an emerging hub in maintenance of genome stability and cancer pathogenesis. Oncogene.

[94] Xuan J., Gitareja K., Brajanovski N., Sanij E. (2021). Harnessing the Nucleolar DNA Damage Response in Cancer Therapy. Genes.

[95] Jayaraman S., Chittiboyina S., Bai Y., Abad P.C., Vidi P.A., Stauffacher C.V., Lelievre S.A. (2017). The nuclear mitotic apparatus protein NuMA controls rDNA transcription and mediates the nucleolar stress response in a p53-independent manner. Nucleic Acids Res..

[96] Russo A., Russo G. (2017). Ribosomal Proteins Control or Bypass p53 during Nucleolar Stress. Int. J. Mol. Sci..

[97] Juan G., Cordon-Cardo C. (2001). Intranuclear compartmentalization of cyclin E during the cell cycle: Disruption of the nucleoplasm-nucleolar shuttling of cyclin E in bladder cancer. Cancer Res..

[98] Liu J., Hebert M.D., Ye Y., Templeton D.J., Kung H., Matera A.G. (2000). Cell cycle-dependent localization of the CDK2-cyclin E complex in Cajal (coiled) bodies. J. Cell Sci..

[99] Hu J., Cai X.F., Yan G. (2009). Alphavirus M1 induces apoptosis of malignant glioma cells via downregulation and nucleolar translocation of p21WAF1/CIP1 protein. Cell Cycle.

[100] Hu B., Hua L., Ni W., Wu M., Yan D., Chen Y., Lu C., Chen B., Wan C. (2017). Nucleostemin/GNL3 promotes nucleolar polyubiquitylation of p27(kip1) to drive hepatocellular carcinoma progression. Cancer Lett..

[101] Bhaskaran N., van Drogen F., Ng H.F., Kumar R., Ekholm-Reed S., Peter M., Sangfelt O., Reed S.I. (2013). Fbw7alpha and Fbw7gamma collaborate to shuttle cyclin E1 into the nucleolus for multiubiquitylation. Mol. Cell Biol..

[102] Welcker M., Orian A., Grim J.E., Eisenman R.N., Clurman B.E. (2004). A nucleolar isoform of the Fbw7 ubiquitin ligase regulates c-Myc and cell size. Curr. Biol..

[103] Sanders J.A., Gruppuso P.A. (2005). Nucleolar localization of hepatic c-Myc: A potential mechanism for c-Myc regulation. Biochim. Biophys. Acta.

[104] Michishita E., Park J.Y., Burneskis J.M., Barrett J.C., Horikawa I. (2005). Evolutionarily conserved and nonconserved cellular localizations and functions of human SIRT proteins. Mol. Biol. Cell.

[105] Tasselli L., Zheng W., Chua K.F. (2017). SIRT6: Novel Mechanisms and Links to Aging and Disease. Trends Endocrinol. Metab..

[106] Tang B.L. (2015). SIRT7 and hepatic lipid metabolism. Front. Cell Dev. Biol..

[107] Kreiner G., Sonmez A., Liss B., Parlato R. (2019). Integration of the Deacetylase SIRT1 in the Response to Nucleolar Stress: Metabolic Implications for Neurodegenerative Diseases. Front. Mol. Neurosci..

[108] Rieker C., Engblom D., Kreiner G., Domanskyi A., Schober A., Stotz S., Neumann M., Yuan X., Grummt I., Schutz G. (2011). Nucleolar disruption in dopaminergic neurons leads to oxidative damage and parkinsonism through repression of mammalian target of rapamycin signaling. J. Neurosci..

[109] Frottin F., Schueder F., Tiwary S., Gupta R., Korner R., Schlichthaerle T., Cox J., Jungmann R., Hartl F.U., Hipp M.S. (2019). The nucleolus functions as a phase-separated protein quality control compartment. Science.

[110] Azkanaz M., Lopez A.R., de Boer B., Huiting W., Angrand P.O., Vellenga E., Kampinga H.H., Bergink S., Martens J.H., Schuringa J.J. (2019). Protein quality control in the nucleolus safeguards recovery of epigenetic regulators after heat shock. Elife.

[111] Amer-Sarsour F., Ashkenazi A. (2019). The Nucleolus as a Proteostasis Regulator. Trends Cell Biol..

[112] Golstein P. (2017). Conserved nucleolar stress at the onset of cell death. FEBS J..

[113] Zhang W., Cheng W., Parlato R., Guo X., Cui X., Dai C., Xu L., Zhu J., Zhu M., Luo K. (2020). Nucleolar stress induces a senescence-like phenotype in smooth muscle cells and promotes development of vascular degeneration. Aging.

[114] Kalita K., Makonchuk D., Gomes C., Zheng J.J., Hetman M. (2008). Inhibition of nucleolar transcription as a trigger for neuronal apoptosis. J. Neurochem..

[115] James A., Wang Y., Raje H., Rosby R., DiMario P. (2014). Nucleolar stress with and without p53. Nucleus.

[116] Donati G., Brighenti E., Vici M., Mazzini G., Trere D., Montanaro L., Derenzini M. (2011). Selective inhibition of rRNA transcription downregulates E2F-1: A new p53-independent mechanism linking cell growth to cell proliferation. J. Cell Sci..

[117] Iadevaia V., Caldarola S., Biondini L., Gismondi A., Karlsson S., Dianzani I., Loreni F. (2010). PIM1 kinase is destabilized by ribosomal stress causing inhibition of cell cycle progression. Oncogene.

[118] Dai M.S., Arnold H., Sun X.X., Sears R., Lu H. (2007). Inhibition of c-Myc activity by ribosomal protein L11. EMBO J..

[119] Lee H.C., Wang H., Baladandayuthapani V., Lin H., He J., Jones R.J., Kuiatse I., Gu D., Wang Z., Ma W. (2017). RNA Polymerase I Inhibition with CX-5461 as a Novel Therapeutic Strategy to Target MYC in Multiple Myeloma. Br. J. Haematol..

[120] Gupta S., Santoro R. (2020). Regulation and Roles of the Nucleolus in Embryonic Stem Cells: From Ribosome Biogenesis to Genome Organization. Stem Cell Rep..

[121] Ingolia N.T., Lareau L.F., Weissman J.S. (2011). Ribosome profiling of mouse embryonic stem cells reveals the complexity and dynamics of mammalian proteomes. Cell.

[122] Meshorer E., Misteli T. (2006). Chromatin in pluripotent embryonic stem cells and differentiation. Nat. Rev. Mol. Cell Biol..

[123] Savic N., Bar D., Leone S., Frommel S.C., Weber F.A., Vollenweider E., Ferrari E., Ziegler U., Kaech A., Shakhova O. (2014). lncRNA maturation to initiate heterochromatin formation in the nucleolus is required for exit from pluripotency in ESCs. Cell Stem Cell.

[124] Zentner G.E., Balow S.A., Scacheri P.C. (2014). Genomic characterization of the mouse ribosomal DNA locus. Genes Genomes Genet..

[125] Zaidi S.K., Boyd J.R., Grandy R.A., Medina R., Lian J.B., Stein G.S., Stein J.L. (2016). Expression of Ribosomal RNA and Protein Genes in Human Embryonic Stem Cells Is Associated With the Activating H3K4me3 Histone Mark. J. Cell Physiol..

[126] Tsai R.Y., McKay R.D. (2002). A nucleolar mechanism controlling cell proliferation in stem cells and cancer cells. Genes Dev..

[127] Yang A., Shi G., Zhou C., Lu R., Li H., Sun L., Jin Y. (2011). Nucleolin maintains embryonic stem cell self-renewal by suppression of p53 protein-dependent pathway. J. Biol. Chem..

[128] Watanabe-Susaki K., Takada H., Enomoto K., Miwata K., Ishimine H., Intoh A., Ohtaka M., Nakanishi M., Sugino H., Asashima M. (2014). Biosynthesis of ribosomal RNA in nucleoli regulates pluripotency and differentiation ability of pluripotent stem cells. Stem Cells.

[129] Zhang H., Wu Z., Lu J.Y., Huang B., Zhou H., Xie W., Wang J., Shen X. (2020). DEAD-Box Helicase 18 Counteracts PRC2 to Safeguard Ribosomal DNA in Pluripotency Regulation. Cell Rep..

[130] Hayashi Y., Kuroda T., Kishimoto H., Wang C., Iwama A., Kimura K. (2014). Downregulation of rRNA transcription triggers cell differentiation. PLoS ONE.

[131] Tsoi H., Lau T.C., Tsang S.Y., Lau K.F., Chan H.Y. (2012). CAG expansion induces nucleolar stress in polyglutamine diseases. Proc. Natl. Acad. Sci. USA.

[132] Makhale A., Nanayakkara D., Raninga P., Khanna K.K., Kalimutho M. (2021). CX-5461 Enhances the Efficacy of APR-246 via Induction of DNA Damage and Replication Stress in Triple-Negative Breast Cancer. Int. J. Mol. Sci..

[133] Pan M., Wright W.C., Chapple R.H., Zubair A., Sandhu M., Batchelder J.E., Huddle B.C., Low J., Blankenship K.B., Wang Y. (2021). The chemotherapeutic CX-5461 primarily targets TOP2B and exhibits selective activity in high-risk neuroblastoma. Nat. Commun..

[134] Di Micco R., Krizhanovsky V., Baker D., d’Adda di Fagagna F. (2021). Cellular senescence in ageing: From mechanisms to therapeutic opportunities. Nat. Rev. Mol. Cell Biol..

[135] d’Adda di Fagagna F. (2008). Living on a break: Cellular senescence as a DNA-damage response. Nat. Rev. Cancer.

[136] Pathak R.U., Soujanya M., Mishra R.K. (2021). Deterioration of nuclear morphology and architecture: A hallmark of senescence and aging. Ageing Res. Rev..

[137] Pinho M., Macedo J.C., Logarinho E., Pereira P.S. (2019). NOL12 Repression Induces Nucleolar Stress-Driven Cellular Senescence and Is Associated with Normative Aging. Mol. Cell Biol..

[138] Zanotti S., Vanhauwaert S., Van Neste C., Olexiouk V., Van Laere J., Verschuuren M., Van der Meulen J., Mus L.M., Durinck K., Tilleman L. (2021). MYCN-induced nucleolar stress drives an early senescence-like transcriptional program in hTERT-immortalized RPE cells. Sci. Rep..

[139] Nishimura K., Kumazawa T., Kuroda T., Katagiri N., Tsuchiya M., Goto N., Furumai R., Murayama A., Yanagisawa J., Kimura K. (2015). Perturbation of ribosome biogenesis drives cells into senescence through 5S RNP-mediated p53 activation. Cell Rep..

[140] Wu H.C., Rerolle D., Berthier C., Hleihel R., Sakamoto T., Quentin S., Benhenda S., Morganti C., Wu C., Conte L. (2021). Actinomycin D Targets NPM1c-Primed Mitochondria to Restore PML-Driven Senescence in AML Therapy. Cancer Discov..

[141] Drygin D., Lin A., Bliesath J., Ho C.B., O’Brien S.E., Proffitt C., Omori M., Haddach M., Schwaebe M.K., Siddiqui-Jain A. (2011). Targeting RNA polymerase I with an oral small molecule CX-5461 inhibits ribosomal RNA synthesis and solid tumor growth. Cancer Res..

[142] Pfister A.S. (2019). Emerging Role of the Nucleolar Stress Response in Autophagy. Front. Cell Neurosci..

[143] Jung C.H., Jun C.B., Ro S.H., Kim Y.M., Otto N.M., Cao J., Kundu M., Kim D.H. (2009). ULK-Atg13-FIP200 complexes mediate mTOR signaling to the autophagy machinery. Mol. Biol. Cell.

[144] Ryter S.W., Cloonan S.M., Choi A.M. (2013). Autophagy: A critical regulator of cellular metabolism and homeostasis. Mol. Cells.

[145] Katagiri N., Kuroda T., Kishimoto H., Hayashi Y., Kumazawa T., Kimura K. (2015). The nucleolar protein nucleophosmin is essential for autophagy induced by inhibiting Pol I transcription. Sci. Rep..

[146] Dannheisig D.P., Schimansky A., Donow C., Pfister A.S. (2021). Nucleolar Stress Functions Upstream to Stimulate Expression of Autophagy Regulators. Cancers.

[147] Li L., Li Y., Zhao J., Fan S., Wang L., Li X. (2016). CX-5461 induces autophagy and inhibits tumor growth via mammalian target of rapamycin-related signaling pathways in osteosarcoma. Onco Targets Ther..

[148] Okamoto S., Miyano K., Kajikawa M., Yamauchi A., Kuribayashi F. (2020). The rRNA synthesis inhibitor CX-5461 may induce autophagy that inhibits anticancer drug-induced cell damage to leukemia cells. Biosci. Biotechnol. Biochem..

[149] Lafita-Navarro M.C., Conacci-Sorrell M. (2022). Nucleolar stress: From development to cancer. Semin. Cell Dev. Biol..

[150] Parlato R., Kreiner G. (2013). Nucleolar activity in neurodegenerative diseases: A missing piece of the puzzle?. J. Mol. Med..

[151] Hein N., Hannan K.M., George A.J., Sanij E., Hannan R.D. (2013). The nucleolus: An emerging target for cancer therapy. Trends Mol. Med..

[152] Chen S., He H., Wang Y., Liu L., Liu Y., You H., Dong Y., Lyu J. (2018). Poor prognosis of nucleophosmin overexpression in solid tumors: A meta-analysis. BMC Cancer.

[153] Barber M.F., Michishita-Kioi E., Xi Y., Tasselli L., Kioi M., Moqtaderi Z., Tennen R.I., Paredes S., Young N.L., Chen K. (2012). SIRT7 links H3K18 deacetylation to maintenance of oncogenic transformation. Nature.

[154] Okamoto N., Yasukawa M., Nguyen C., Kasim V., Maida Y., Possemato R., Shibata T., Ligon K.L., Fukami K., Hahn W.C. (2011). Maintenance of tumor initiating cells of defined genetic composition by nucleostemin. Proc. Natl. Acad. Sci. USA.

[155] Blank M.F., Grummt I. (2017). The seven faces of SIRT7. Transcription.

[156] Bywater M.J., Poortinga G., Sanij E., Hein N., Peck A., Cullinane C., Wall M., Cluse L., Drygin D., Anderes K. (2012). Inhibition of RNA polymerase I as a therapeutic strategy to promote cancer-specific activation of p53. Cancer Cell.

[157] Pecoraro A., Carotenuto P., Russo G., Russo A. (2019). Ribosomal protein uL3 targets E2F1 and Cyclin D1 in cancer cell response to nucleolar stress. Sci. Rep..

[158] Haddach M., Schwaebe M.K., Michaux J., Nagasawa J., O’Brien S.E., Whitten J.P., Pierre F., Kerdoncuff P., Darjania L., Stansfield R. (2012). Discovery of CX-5461, the First Direct and Selective Inhibitor of RNA Polymerase I, for Cancer Therapeutics. ACS Med. Chem. Lett..

[159] Cornelison R., Biswas K., Llaneza D.C., Harris A.R., Sosale N.G., Lazzara M.J., Landen C.N. (2021). CX-5461 Treatment Leads to Cytosolic DNA-Mediated STING Activation in Ovarian Cancer. Cancers.

[160] Hilton J., Gelmon K., Bedard P.L., Tu D., Xu H., Tinker A.V., Goodwin R., Laurie S.A., Jonker D., Hansen A.R. (2022). Results of the phase I CCTG IND.231 trial of CX-5461 in patients with advanced solid tumors enriched for DNA-repair deficiencies. Nat. Commun..

[161] Khot A., Brajanovski N., Cameron D.P., Hein N., Maclachlan K.H., Sanij E., Lim J., Soong J., Link E., Blombery P. (2019). First-in-Human RNA Polymerase I Transcription Inhibitor CX-5461 in Patients with Advanced Hematologic Cancers: Results of a Phase I Dose-Escalation Study. Cancer Discov..

[162] Lee J., Hwang Y.J., Ryu H., Kowall N.W., Ryu H. (2014). Nucleolar dysfunction in Huntington’s disease. Biochim. Biophys. Acta.

[163] Arogundade O.A., Nguyen S., Leung R., Wainio D., Rodriguez M., Ravits J. (2021). Nucleolar stress in C9orf72 and sporadic ALS spinal motor neurons precedes TDP-43 mislocalization. Acta Neuropathol. Commun..

[164] Pietrzak M., Rempala G., Nelson P.T., Zheng J.J., Hetman M. (2011). Epigenetic silencing of nucleolar rRNA genes in Alzheimer’s disease. PLoS ONE.

[165] Caudle W.M., Kitsou E., Li J., Bradner J., Zhang J. (2009). A role for a novel protein, nucleolin, in Parkinson’s disease. Neurosci. Lett..

[166] Vilotti S., Codrich M., Dal Ferro M., Pinto M., Ferrer I., Collavin L., Gustincich S., Zucchelli S. (2012). Parkinson’s disease DJ-1 L166P alters rRNA biogenesis by exclusion of TTRAP from the nucleolus and sequestration into cytoplasmic aggregates via TRAF6. PLoS ONE.

[167] Lee J., Hwang Y.J., Boo J.H., Han D., Kwon O.K., Todorova K., Kowall N.W., Kim Y., Ryu H. (2011). Dysregulation of upstream binding factor-1 acetylation at K352 is linked to impaired ribosomal DNA transcription in Huntington’s disease. Cell Death Differ..

[168] Sonmez A., Mustafa R., Ryll S.T., Tuorto F., Wacheul L., Ponti D., Litke C., Hering T., Kojer K., Koch J. (2021). Nucleolar stress controls mutant Huntington toxicity and monitors Huntington’s disease progression. Cell Death Dis..

[169] Latonen L. (2011). Nucleolar aggresomes as counterparts of cytoplasmic aggresomes in proteotoxic stress. Proteasome inhibitors induce nuclear ribonucleoprotein inclusions that accumulate several key factors of neurodegenerative diseases and cancer. Bioessays.

[170] Tao Z., Wang H., Xia Q., Li K., Jiang X., Xu G., Wang G., Ying Z. (2015). Nucleolar stress and impaired stress granule formation contribute to C9orf72 RAN translation-induced cytotoxicity. Hum. Mol. Genet..

[171] White M.R., Mitrea D.M., Zhang P., Stanley C.B., Cassidy D.E., Nourse A., Phillips A.H., Tolbert M., Taylor J.P., Kriwacki R.W. (2019). C9orf72 Poly(PR) Dipeptide Repeats Disturb Biomolecular Phase Separation and Disrupt Nucleolar Function. Mol. Cell.

[172] Maina M.B., Bailey L.J., Wagih S., Biasetti L., Pollack S.J., Quinn J.P., Thorpe J.R., Doherty A.J., Serpell L.C. (2018). The involvement of tau in nucleolar transcription and the stress response. Acta Neuropathol. Commun..

[173] Marquez-Lona E.M., Tan Z., Schreiber S.S. (2012). Nucleolar stress characterized by downregulation of nucleophosmin: A novel cause of neuronal degeneration. Biochem. Biophys. Res. Commun..

[174] Herrmann D., Parlato R. (2018). C9orf72-associated neurodegeneration in ALS-FTD: Breaking new ground in ribosomal RNA and nucleolar dysfunction. Cell Tissue Res..

[175] Michel C.I., Holley C.L., Scruggs B.S., Sidhu R., Brookheart R.T., Listenberger L.L., Behlke M.A., Ory D.S., Schaffer J.E. (2011). Small nucleolar RNAs U32a, U33, and U35a are critical mediators of metabolic stress. Cell Metab..

[176] Schaffer J.E. (2020). Death by lipids: The role of small nucleolar RNAs in metabolic stress. J. Biol. Chem..

[177] Dupuis-Sandoval F., Poirier M., Scott M.S. (2015). The emerging landscape of small nucleolar RNAs in cell biology. Wiley Interdiscip. Rev. RNA.

[178] Falaleeva M., Pages A., Matuszek Z., Hidmi S., Agranat-Tamir L., Korotkov K., Nevo Y., Eyras E., Sperling R., Stamm S. (2016). Dual function of C/D box small nucleolar RNAs in rRNA modification and alternative pre-mRNA splicing. Proc. Natl. Acad. Sci. USA.

[179] Bratkovic T., Rogelj B. (2014). The many faces of small nucleolar RNAs. Biochim. Biophys. Acta.

[180] Holley C.L., Li M.W., Scruggs B.S., Matkovich S.J., Ory D.S., Schaffer J.E. (2015). Cytosolic accumulation of small nucleolar RNAs (snoRNAs) is dynamically regulated by NADPH oxidase. J. Biol. Chem..

[181] Lee J., Harris A.N., Holley C.L., Mahadevan J., Pyles K.D., Lavagnino Z., Scherrer D.E., Fujiwara H., Sidhu R., Zhang J. (2016). Rpl13a small nucleolar RNAs regulate systemic glucose metabolism. J. Clin. Investig..

[182] Wu J., Jiang X., Li Y., Zhu T., Zhang J., Zhang Z., Zhang L., Zhang Y., Wang Y., Zou X. (2018). PHA-4/FoxA senses nucleolar stress to regulate lipid accumulation in Caenorhabditis elegans. Nat. Commun..

[183] Yoshizawa T., Karim M.F., Sato Y., Senokuchi T., Miyata K., Fukuda T., Go C., Tasaki M., Uchimura K., Kadomatsu T. (2014). SIRT7 controls hepatic lipid metabolism by regulating the ubiquitin-proteasome pathway. Cell Metab..

[184] Shin J., He M., Liu Y., Paredes S., Villanova L., Brown K., Qiu X., Nabavi N., Mohrin M., Wojnoonski K. (2013). SIRT7 represses Myc activity to suppress ER stress and prevent fatty liver disease. Cell Rep..

[185] Ryu D., Jo Y.S., Lo Sasso G., Stein S., Zhang H., Perino A., Lee J.U., Zeviani M., Romand R., Hottiger M.O. (2014). A SIRT7-dependent acetylation switch of GABPbeta1 controls mitochondrial function. Cell Metab..

[186] Mohrin M., Shin J., Liu Y., Brown K., Luo H., Xi Y., Haynes C.M., Chen D. (2015). Stem cell aging. A mitochondrial UPR-mediated metabolic checkpoint regulates hematopoietic stem cell aging. Science.

[187] Liu Y., He Y., Jin A., Tikunov A.P., Zhou L., Tollini L.A., Leslie P., Kim T.H., Li L.O., Coleman R.A. (2014). Ribosomal protein-Mdm2-p53 pathway coordinates nutrient stress with lipid metabolism by regulating MCD and promoting fatty acid oxidation. Proc. Natl. Acad. Sci. USA.

[188] Guarente L. (1997). Link between aging and the nucleolus. Genes Dev..

[189] Kennedy B.K., Gotta M., Sinclair D.A., Mills K., McNabb D.S., Murthy M., Pak S.M., Laroche T., Gasser S.M., Guarente L. (1997). Redistribution of silencing proteins from telomeres to the nucleolus is associated with extension of life span in S. cerevisiae. Cell.

[190] Tiku V., Jain C., Raz Y., Nakamura S., Heestand B., Liu W., Spath M., Suchiman H.E.D., Muller R.U., Slagboom P.E. (2017). Small nucleoli are a cellular hallmark of longevity. Nat. Commun..

[191] Buchwalter A., Hetzer M.W. (2017). Nucleolar expansion and elevated protein translation in premature aging. Nat. Commun..

[192] Gensous N., Ravaioli F., Pirazzini C., Gramignoli R., Ellis E., Storci G., Capri M., Strom S., Laconi E., Franceschi C. (2020). Aging and Caloric Restriction Modulate the DNA Methylation Profile of the Ribosomal RNA Locus in Human and Rat Liver. Nutrients.

[193] Smith D.L., Li C., Matecic M., Maqani N., Bryk M., Smith J.S. (2009). Calorie restriction effects on silencing and recombination at the yeast rDNA. Aging Cell.

[194] Wang M., Lemos B. (2019). Ribosomal DNA harbors an evolutionarily conserved clock of biological aging. Genome Res..

[195] Morlot S., Song J., Leger-Silvestre I., Matifas A., Gadal O., Charvin G. (2019). Excessive rDNA Transcription Drives the Disruption in Nuclear Homeostasis during Entry into Senescence in Budding Yeast. Cell Rep..

[196] Perry R.P. (1966). Nucleolus: Structure and function. Science.

[197] Derenzini M., Trere D., Pession A., Govoni M., Sirri V., Chieco P. (2000). Nucleolar size indicates the rapidity of cell proliferation in cancer tissues. J. Pathol..

[198] Hariharan N., Sussman M.A. (2014). Stressing on the nucleolus in cardiovascular disease. Biochim. Biophys. Acta.

[199] Calo E., Gu B., Bowen M.E., Aryan F., Zalc A., Liang J., Flynn R.A., Swigut T., Chang H.Y., Attardi L.D. (2018). Tissue-selective effects of nucleolar stress and rDNA damage in developmental disorders. Nature.

[200] Bianco C., Mohr I. (2019). Ribosome biogenesis restricts innate immune responses to virus infection and DNA. Elife.

[201] Matos-Perdomo E., Machin F. (2019). Nucleolar and Ribosomal DNA Structure under Stress: Yeast Lessons for Aging and Cancer. Cells.

[202] Parlato R., Liss B. (2014). How Parkinson’s disease meets nucleolar stress. Biochim. Biophys. Acta.

[203] Latonen L. (2019). Phase-to-Phase With Nucleoli—Stress Responses, Protein Aggregation and Novel Roles of RNA. Front. Cell Neurosci..

[204] Avitabile D., Bailey B., Cottage C.T., Sundararaman B., Joyo A., McGregor M., Gude N., Truffa S., Zarrabi A., Konstandin M. (2011). Nucleolar stress is an early response to myocardial damage involving nucleolar proteins nucleostemin and nucleophosmin. Proc. Natl. Acad. Sci. USA.

[205] Pfister J.A., D’Mello S.R. (2015). Insights into the regulation of neuronal viability by nucleophosmin/B23. Exp. Biol. Med..

[206] Zhang B., Wang H., Jiang B., Liang P., Liu M., Deng G., Xiao X. (2010). Nucleolin/C23 is a negative regulator of hydrogen peroxide-induced apoptosis in HUVECs. Cell Stress Chaperones.

